# Understanding Food and Nutrition Insecurity Among College Students: Evidence from a Cross-Campus Study

**DOI:** 10.3390/nu18060951

**Published:** 2026-03-18

**Authors:** Kritee Niroula, Summaya Abdul Razak, Jolaade Kalinowski, Loneke T. Blackman Carr, Amy Gorin, Kristen Cooksey Stowers

**Affiliations:** 1 Department of Allied Health Sciences, University of Connecticut, Storrs, CT 06269, USA; ubl24004@uconn.edu (K.N.); summaya.abdul_razak@uconn.edu (S.A.R.); 2 Department of Human Development and Family Sciences, University of Connecticut, Storrs, CT 06269, USA; jolaade.kalinowski@uconn.edu; 3 Department of Nutritional Sciences, University of Connecticut, Storrs, CT 06269, USA; loneke.blackman_carr@uconn.edu; 4 Department of Psychological Sciences, University of Connecticut, Storrs, CT 06269, USA; amy.gorin@uconn.edu; 5 Rudd Center for Food Policy and Health, University of Connecticut, Hartford, CT 06103, USA

**Keywords:** food insecurity, nutrition insecurity, higher education, housing insecurity, university students

## Abstract

Background: Food insecurity is defined as having limited access to food, while nutrition insecurity is characterized as a lack of consistent access to affordable and acceptable foods that support health, manage or prevent disease, and meet daily nutritional needs. College students face increased risks of food and nutrition insecurity, yet the issue is understudied. This study examined the patterns of food and nutrition insecurity among students at a public university across main and regional campuses. Methods: We conducted a cross-sectional survey using Qualtrics for participant recruitment in November 2023. The USDA’s 10-item toolFood insecurity was measured using the USDA’s 10-item tooland housing security was measured using the U.S. Census Bureau’s National Survey of Income and Program Participation 6-item tool. We used ANOVAs and logistic regression to examine differences across demographics. Data analysis was done using SPSS version 29. Results: There were 6538 student responses. Of these, 36% of students were food insecure, while 20% were nutritionally insecure. Comparatively, food and nutrition insecurity were significantly higher among students with low-income (*p* < 0.001), housing instability (*p* < 0.001), a higher number of dependents, and those indicating that they were single/unmarried (*p* = 0.005), first-generation (*p* < 0.001), and Pell grant eligible (*p* < 0.001). Annual income and housing security emerged as significant predictors: lower income was approximately twice as likely to be associated with food insecurity, while those reporting housing insecurity were six times more likely to experience food insecurity. Conclusions: The study findings reveal disparities in food and nutrition insecurity among a diverse student population at a public university. Addressing the issue among them is crucial and requires a multifaceted, inclusive approach. Emergency financial assistance and structural interventions that promote housing security are warranted.

## 1. Introduction

Food insecurity has emerged as a significant concern in the US. The US Department of Agriculture (USDA) reported a 13.5% prevalence of food insecurity in 2023, up from 12.8% in 2022 [1]. Individuals facing food insecurity have limited access to sufficient and healthy food, and often run out of food, forcing them to prioritize food over other basic needs [2]. For individuals experiencing food insecurity, affordable food items are often highly processed and high in sugar, salt, and saturated fat [2]. The quality and variety of available foods are compromised, leading to overconsumption and contributing to overweight and obesity [3]. The concept of nutrition security emerged consistently over the years, as the focus shifted from quantity to quality [3]. While food security refers to the quantity of food, nutrition security highlights the nutritional quality, affordability, and accessibility that promote overall well-being [4].

Food insecurity is widespread among college and university students, posing significant challenges to their access to safe, nutritious food [5]. A growing body of evidence reports its prevalence between 20 and 50%, exceeding the national average by approximately 12% [6,7,8]. Students who identify as first-generation, Pell grant recipients come from families with lower income and belong to racial or ethnic minorities who are at a higher risk of food insecurity compared to the non-first-generation students, those from higher income households, and the majority racial backgrounds [9]. In general, many university students in the US have been found to experience food insecurity for several reasons, including insufficient financial resources, housing instability, a lack of transportation and grocery stores within an accessible distance, and a lack of cooking skills [10]. The costs of obtaining a college degree have rapidly increased over the years [11]. While some students can receive federal and other sources of financial aid, these funds often do not cover essential living costs, including food. The Pell Grant, which used to cover almost 80% of the costs to get a college degree, now covers just about 31% of total costs. The intensified economic instability forces them to work multiple jobs or often choose between work and classes [11]. Moreover, recent changes to federal student aid, such as reduced funds for graduate students, loans unavailable to new borrowers, and few repayment options, are forcing students to take out private loans for higher education, which typically have higher interest rates [12,13].

Empirical evidence indicates that students may experience anxiety and chronic stress when handling both their finances and dietary needs, thus leading to a cumulative effect on their ability to thrive academically and socially [14]. Students reported difficulty concentrating, lower grade-point averages, and dropping courses [15,16]. Other psychological issues as a result of food insecurity included hopelessness and a lack of confidence to seek help [10]. In addition to tuition, living expenses such as food, monthly rent, and college supplies are also adding to the financial burden [17]. A national survey of college students found that only about 20% of food-insecure students received Supplemental Nutrition Assistance Program (SNAP) benefits [17]. Those who met the eligibility criteria did not participate in the program due to challenges in navigating the application process [18].

Hunger-free policies are adopted by college campuses through initiatives such as meal programs and food pantries to address immediate needs. However, limited evidence exists on their effectiveness and reach [19]. The location and service hours of college food pantries often made these resources inaccessible [20]. Given that college students are at higher risk of food insecurity, this study aimed to explore the food and nutrition security of undergraduate, graduate, medical, dental, and law students across a large public university in the Northeast’s main and regional campuses and associated sociodemographic factors.

### Study Purpose

The goal of this study was to examine food and nutrition security among graduate and undergraduate students across the seven campuses of a large public university in the Northeast. The specific aims of the study were as follows:

(a) Assess the prevalence of food insecurity among students at each of a large public Northeastern university’s campuses.
(b) Assess the prevalence of nutrition security among students at each of a large public Northeastern university’s campuses.
(c) Examine the relationships among food and nutrition insecurity, housing security, annual income, employment, race, gender, and other demographics.

## 2. Materials and Methods

### 2.1. Study Design

The prevalence of food and nutrition security among students enrolled at a large, public Northeastern university was assessed using a cross-sectional survey. Data was collected during the fall semester of 2023 and included all seven geographically diverse campuses (Storrs, Avery Point, Hartford, Waterbury, Law School, UConn Health and Stamford). The study protocol was reviewed and approved by the university’s Institutional Review Board (IRB Protocol: H20-0173). The survey included questions on food insecurity, nutrition insecurity, housing insecurity, and other aspects of food access and resources on campus.

### 2.2. Sample and Inclusion Criteria

The study included all students from all campuses who were enrolled as degree-seeking graduate and undergraduate students, as well as medical, dental, or law students at the time of data collection.

#### Sample Size

The sample size (*n*) was determined using a standard formula for cross-sectional studies estimating prevalence [21].

$$n=\frac{z^{2}\;p(1-p)}{d^{2}}$$

Based on published estimates indicating a college food insecurity prevalence (*p*) of 41%, z corresponding to a 95% confidence level, and a precision of 2.5% (d), the minimum required sample size was calculated to be 1486. To improve representation across campuses and student subpopulations, the sample size was increased to 2500, which is about 8% of the students currently enrolled at all the university’s campuses. As this was a cross-sectional study, attrition was not applicable.

### 2.3. Study Recruitment

The survey was administered using Qualtrics XM Platform (https://www.qualtrics.com, Qualtrics, Provo, UT, USA). To recruit participants, each campus administrative office disseminated the survey link and study information through campus listservs, social media, and in-class announcements. All students enrolled at university campuses received an email inviting them to participate in the survey, and the survey platform was designed to collect responses anonymously. To enhance representativeness, electronic recruitment efforts were complemented with in-person outreach at each campus location.

Students were able to log into the survey site using their university NetIDs. Only enrolled students could log in and participate. Upon accessing the survey link, they were provided information about the study’s purpose, associated risks, and benefits. They had to provide consent by selecting “Yes, I consent to participate in this study” at the bottom of the form. If they selected “No, I do not participate in this study,” the survey ended. The survey took between 15 and 20 min to complete. Participants who completed the survey were entered into a raffle drawing, with 60 winners receiving $25 Amazon gift cards.

### 2.4. Survey Instruments

Food and nutrition security instruments capture experiences that are connected yet separate. Food insecurity measures reflect insufficient access to food, followed by concerns about running out of food, skipping meals, and reducing portion sizes [22]. On the other hand, nutrition security concerns diet quality and the health consequences of the foods available. Together, these tools capture the quality and quantity aspects of food [22,23]. Additional details about each measure are outlined below.

Food insecurity: The validated 10-item food security module from the U.S. Department of Agriculture (USDA) was used. The questions asked whether they would run out of food, did not have money to buy more, and could not afford balanced meals [22].

Nutrition Security: The tool consisted of 4 items adapted from the Household Nutrition Security survey, a validated instrument with a Cronbach’s alpha of 0.85. The survey questions investigated whether participants could not get healthy food, had to eat the same food for several days in a row, or had to eat food that was not good for their health and well-being [23]. Food and nutrition security instruments capture experiences that are connected yet separate.

Housing Security: Housing security was assessed using a 6-item tool measure previously used by Robbins et al. [24]. The measure was administered in its original form, with no modifications to the items or response options. These questions were modified from the National Survey of Income and Program Participation (SIPP) from the United States Census Bureau and were used to assess housing insecurity status. The questions asked if the participants did not pay or underpaid their rent or mortgage, moved two or more times, moved in with other people, or did not pay their utility bills [24].

Demographics: The university linked survey responses to the questions outlined above to the following student demographic information via NetIDs and university administrative records: age, sex, race/ethnicity, first-generation status, Pell eligibility, household size, number of dependents, and employment status. The university then shared the dataset with the research team as a fully deidentified dataset, securing accurate classification of student characteristics while maintaining participant confidentiality.

### 2.5. Statistical Analysis

Analyses were computed using SPSS version 29 (IBM Corp., Armonk, NY, USA). Participants’ demographic characteristics were reported using frequencies and responses. The food security tool calculated a total raw score for each participant, ranging from 0 to 10. Based on the following score cut-offs, they were divided into four categories of food security: A score of 0 signified high food security, reflecting no reported problems or restrictions in food access. Scores of 1–2 represented marginal food security, characterized by concerns about running out of food but without substantial changes in food intake. Scores of 3–5 indicated low food security, marked by decreased food quality or variety. Scores of 6–10 reflected very low food security, the most severe level, involving disrupted eating patterns and decreased food intake. As stated by the USDA, the high and marginal categories are further classified as food secure, while the low and very low categories are classified as food insecure [22].

The household nutrition security tool from the Gretchen Swanson Center for Nutrition, similar to the food insecurity tool, generated a total score for each respondent. An average score of ≤2 was categorized as nutrition insecurity and >2 as nutrition security [23]. Housing security was measured using six questions, with responses of “yes” or “no.” An affirmative response to any of the questions was considered housing-insecure [24].

Participants’ demographics were computed using frequencies and percentages. To study differences in food and nutrition insecurity across groups, *t*-tests and Analysis of Variance (ANOVA) were used to calculate means, standard deviations (SDs), and their respective *p*-values. Logistic regression models were also performed individually to find potential predictors of food and nutrition insecurity, with results reported as adjusted odds ratios and their 95% confidence intervals (CIs). Each model included age, gender, race, marital status, employment status, Pell grant eligibility, number of dependents, annual income, housing security, and first-generation student status as predictors. Statistical significance was determined at *p* < 0.05.

## 3. Results

### 3.1. Participants’ Demographics

A total of 6538 responses were recorded. The mean age was 22.01 ± 6.09 years with a range of 16–78 years. Compared to the overall university population given by the Fall 2023 Student Enrollment Census (*n* = 32,332) [25,26,27], the sample closely mirrors institutional demographics in several key areas. In the survey sample, undergraduate students comprised 73.7%, and graduate students 20.1%, with additional representation from professional programs, including Law (2.2%), Medicine (1.5%), Dental Medicine (0.8%) and Pharmacy (0.3%). The university’s population data indicate that undergraduate students represent 74.2%, followed by graduate students (20.1%), Law (1.7%), Medicine (1.4%), Dental Medicine (0.6%) and Pharmacy (0.4%). More information on race and gender is presented in Table 1 below.

In addition to that, almost half of the respondents (49.4%) had an annual income between 0 and $5000, and 32% had a meal plan. More details of the participants’ sociodemographic characteristics are available in Table 2.

### 3.2. Food and Nutrition Insecurity Mean Scores Across Demographics

Overall, 2347 (35.9%) of the students experienced food insecurity, with 15.7% experiencing low food security and 20.2% experiencing very low food security. In addition, 19.8% were considered nutritionally insecure.

Table 3 includes the mean food and nutrition insecurity scores across sociodemographic groups. Single participants, or those who were not married, exhibited higher food insecurity (*p* = 0.005) and nutrition insecurity (*p* < 0.001) compared to those who were married or in a domestic partnership. Students who reported annual income less than $20,000, i.e., less than 5000, 10,000–15,000, and 15,000–20,000, markedly experienced higher food insecurity than those earning more than $20,000 (*p* < 0.001). In addition, similar significant differences were observed among lower-income students who exhibited higher levels of nutrition insecurity (*p* < 0.001). Students supporting a greater number of dependents exhibited similar patterns, reporting greater food insecurity (*p*-values were 0.006 and 0.025). Furthermore, the group of participants who were Pell Grant-eligible and first-generation students demonstrated significant vulnerability across both food and nutrition insecurity (*p* < 0.001). In addition, students experiencing housing insecurity suffered heightened food and nutrition insecurity compared to housing-secure participants (*p* < 0.001). Conversely, employed participants reported higher food and nutrition insecurity than unemployed participants (*p* < 0.001).

### 3.3. Risk Estimates Across Demographics

Table 4 presents the adjusted odds ratios with predictors from the regression analysis. Housing security was significant across both models, with students reporting an unstable housing situation more than six times as likely to experience food insecurity and over four times as likely to experience nutrition insecurity compared to students with secure housing circumstances (*p* < 0.001). Annual income emerged as another significant predictor for both food and nutrition insecurity. Students having an annual income lower than $20,000, categorized as less than $5000, between $5000 and 10,000, and $10,000–20,000, had elevated odds of experiencing both food and nutrition insecurity, ranging from 1.52 to 1.99. First-generation students were nearly twice as likely to experience both food and nutrition insecurity compared to students who did not belong to the first-generation category (*p* < 0.001). Similarly, Pell-eligible students experienced more than 2-fold higher food insecurity than non-eligible students (*p* < 0.001).

On the contrary, race was also significantly associated with both outcomes. In comparison to Non-Resident Alien (NRA) students, White students were approximately half as likely to experience food (*p* < 0.001) and nutrition insecurity (*p* = 0.003), while Latino and Black students exhibited higher odds of food insecurity, with their respective values being 1.24 and 1.56 (*p* < 0.001).

## 4. Discussion

This study found evidence of heightened food and nutrition insecurity among students across campuses at a large, public university. While much of the existing literature focuses primarily on traditional graduate and undergraduate students, this study captured the experiences of a wide range of learners, including medical, dental, and law students, as well as non-degree and pharmacy students. Results across both food and nutrition insecurity among university students showed significant associations with socioeconomic and demographic characteristics. The differences in scores demonstrated that single participants, Pell-eligible first-generation students, and those in unstable housing conditions, such as not paying or underpaying their rent or mortgage or moving in with other people due to financial problems, consistently reported higher levels of food insecurity.

Notably, annual income emerged as a key predictor of food and nutrition insecurity in this study. The trend documented an interaction of financial burden and social disadvantage in widening the gap in college food insecurity. Constrained financial resources prevent students from purchasing nutritious and balanced meals [28]. An increase in the monthly food allowance has been associated with increased purchases of nutritious foods [29]. Similarly, those who were single struggled with food insecurity more than married participants or those in a domestic partnership. The findings were consistent with prior research indicating that married participants were less vulnerable to both food and nutritional insecurity [30]. The possible reason could be an improved financial situation due to increased income, pooled financial resources, and shared everyday expenses [30]. Students experiencing food insecurity might be more likely to seek employment to meet their basic food needs. A student basic needs survey conducted at the University of Arizona in the Spring of 2021 revealed that employed students reported experiencing food insecurity [31]. Similar results were reported from two separate studies conducted at universities in the Midwestern and Southeastern regions of the US where employed students were more likely to experience low or very low food security [24,32]. It has also been reported that students working more hours to support themselves financially had limited opportunities to access available meals [33]. It is also known that employment does not necessarily confer eligibility for federal nutrition assistance like SNAP. Under current SNAP regulations, students enrolled at least half-time must meet specific exemption criteria to qualify, including working a minimum of 20 h per week, participating in a state or federally funded work study program, caring for a dependent, or meeting other eligibility requirements. Students who are employed but do not consistently meet the 20 h threshold may therefore remain ineligible for SNAP benefits [34]. These structural ineligibility requirements may help explain the observed association between employment and food insecurity and have important implications for assessing the impact of SNAP’s work requirements for Able-Bodied Adults without Dependents (ABAWDs) which most college students are classified as. Notably, in 2023 when the survey was administered, there was a statewide waiver exempting ABAWDS from the 20 h per week work rule.

Participants supporting multiple household members were more likely to experience food insecurity due to higher financial obligations [35]. Similarly, the study reported increased burden of both food and nutrition insecurity among first-generation students. They receive limited financial support from family and guidance on financial decision-making [36]. Housing security was also found to be significantly associated with both food and nutrition insecurity. Those students experiencing unstable housing were at the highest risk of experiencing food and nutrition insecurity, compromising their access to adequate, nutritious foods. Among a sample of Australian university students, food insecurity was closely linked to housing-related vulnerability, particularly among those who were renting or living in shared accommodation [37]. It was also evident among students in Paris, as those living in collective housing or flat sharing were more likely to experience insecurity [38]. Prioritizing rent and monthly utilities leaves them with less budget for food procurement and thus forces them to rely on low-cost, less nutritious options [39,40,41].

The regression findings highlight racial disparities in both food and nutrition insecurity, suggesting the outcomes could be shaped not just by housing or economic status but also by structural racism. A study between 2015 and 2019 reported that food insecurity was more prevalent among Black and Hispanic students, affecting more than 21% of Black students and 26% of Hispanic students, compared to 9% White students [42]. Students from marginalized groups often face compounded challenges related to financial support, familial wealth disparities, institutional discrimination, and access to basic need resources, which can increase their risk of facing food and nutrition insecurity while enrolled in college [43,44].

Food insecurity is highly prevalent among students who are financially dependent on their parents. Because racial and ethnic groups and Black, Hispanic, or Latino students are less likely to receive intergenerational wealth and support for higher education, the racial wealth gap limits their access to food or housing [45]. Psychosocial stressors, inclusive of perceived discrimination and social isolation paired with limited social support, may further contribute to financial strain and reduced access to coping resources [44].

Our results indicated that the prevalence of food insecurity is disproportionate across age groups, marital status, and economic status. To address these challenging factors that put students at increased risk of food and nutrition insecurity, campus food pantries have been considered a resource for “critical emergency relief” [19]. While the approach seems promising, students have reported concerns, such as a lack of transportation and hours of operation, that prevent them from receiving the service [46]. Some others have refrained from using food assistance services because of the stigma associated with appearing poor and needy [46]. Researchers need to prioritize qualitative inquiry, such as one-on-one interviews, to understand experiences, including barriers and solutions to access and utilization [47]. Even though there are existing resources, not all students may have the knowledge to locate and access food sources on campus. For instance, first-year and incoming students may not be aware of the eligibility or the enrollment process. Campus administration should ensure dedicated staff are hired to prioritize communication about existing resources, services, and eligibility, and to provide flexibility in hours of operation [48].

On-campus food resources with ample access to healthy options could be one solution to address food insecurity [49,50]. Dealing with food insecurity requires multi-level approaches to improve economic conditions and create a diverse food environment, ensuring affordable, healthy, safe, and sustainable food options [51]. Collaboration with food banks or other state-level food resources could be a strategy to improve food procurement and enable food pantries to serve students experiencing food insecurity better. Other options may include discounted meal plans or voucher programs for use at the university dining facilities, as well as federal food assistance programs. University administrations can also partner with nearby grocery stores to offer students discounted food. The trends in food and nutrition insecurity are deeply rooted in structural barriers, and efforts to bring about change should focus on policy systems and interventions at the institutional, state, and national levels rather than just providing individual aid.

## 5. Strengths and Limitations

The study possesses several noteworthy strengths. A strength of this study is the large and diverse sample of students across multiple sociodemographically and geographically diverse campuses at a large, public university in the northeastern region., Further, the samples encompasses undergraduate and graduate students, as well as pharmacy, medical, dental, and law students from a wide range of academic programs. Multiple demographic determinants were incorporated into the survey, allowing for a more comprehensive picture of the factors that predict food and nutrition insecurity. This study provides insights into nutrition insecurity among college studies as an understudied population within this emerging topic Exploring nutrition security among college students is critical as it extends beyond traditional food insecurity, and cultivates overall well-being.

Still, there are also some limitations to be acknowledged. Voluntary participation could have increased the risk of under-representation of students experiencing higher levels of food and nutrition insecurity. There is also limited participation by certain subgroups, e.g., Native Americans, which may limit the stability of estimates. Additionally, married students and those living in households with three or more dependents are underrepresented in our sample. Because this study used a cross-sectional design, it does not help determine cause-and-effect relationships; however, the research findings highlight important directions for future longitudinal and mixed-methods research.

As recruitment relied heavily on university communication and outreach, participation might have been more appealing to students experiencing food and nutrition insecurity, potentially leading to higher estimates than would have been observed in a broader student population. To minimize the potential bias, we complemented electronic outreach with in-person and student-led recruitment in high-traffic campus locations. In addition, participants were offered entry into a raffle to promote participation regardless of food security status.

A strength of the study is that it is a large sample representing seven diverse campuses across the state, which vary in key factors such as urbanicity, median income, and food environment. Although the sample is large, it is limited to the students of a single public institution, potentially limiting the generalizability of the findings to other academic institutions, particularly private institutions. Still, across the United States, there are many public, land-grant institutions with regional campuses.

## 6. Conclusions

The findings from this study highlight food and nutrition security as a significant concern among multiple geographically diverse campuses of a large public Northeastern university. Results demonstrate that food and nutrition insecurity are deeply intertwined with broader structural conditions, particularly housing instability, household size, and financial strain. While emergency food assistance plays a critical role in addressing immediate food needs, it cannot mitigate food and nutrition insecurity without structural support. Sustainable solutions require interventions that integrate food and housing security efforts, such as coordinated enrollment in housing assistance and nutrition programs, cross-sector partnerships between housing authorities and food systems organizations, and policy approaches that address the rising cost of housing alongside food access and affordability. Investments in stable housing and access to nutrition assistance programs are essential components of long-term food security and improved health outcomes. Additionally, institutional policies and strategies, including more flexible and affordable meal plans, SNAP enrollment and eligibility, and financial aid to cover students’ basic living and tuition costs, might help reduce immediate food insecurity. Integration of such policies with existing emergency food aid and campus resources could promote sustainability in food and nutrition security.

## Figures and Tables

**Table 1:** Representativeness of survey sample relative to university student population.

	Survey Sample *n* (%)	University Population *n* (%)
Academic Program		
Dental Medicine	52 (0.8)	204 (0.6)
Graduate	1311 (20.1)	6512 (20.1)
Law	145 (2.2)	534 (1.7)
Law LLM	17 (0.3)	-
Medicine	101 (1.5)	449 (1.4)
Non-Degree	62 (0.9)	483 (1.5)
Pharmacy	17 (0.3)	143 (0.4)
Agriculture	13 (0.2)	-
Undergraduate	4820 (73.7)	24,007 (74.2)
Gender		
Female	4135 (63.2)	16,869 (52.2)
Male	2403 (36.8)	14,810 (45.8)
Race		
Non-Resident American	743 (11.5)	3570 (11.0)
Latino	1050 (16.2)	5085 (15.7)
Native American	4 (0.06)	-
Asian American	852 (13.2)	3668 (11.3)
Black	464 (7.2)	2462 (7.6)
Hawaiian/Pacific Islander	1 (0.01)	13 (0.04)
White	3089 (47.8)	15,191 (46.9)
Multi-racial	262 (4.1)	1293 (4.0)

**Table 2:** Sociodemographics among study participants.

Demographics	*n* (%)
Marital Status	
Single/not married	5883 (91.5)
Married or Domestic Partnership	547 (8.5)
Age (years)	
18–30	6067 (92.8)
31–40	314 (4.8)
41 and above	157 (2.4)
Employment status	
No	2867 (44.0)
Yes	3478 (53.2)
Housing Security	
Secure	1924 (61.0)
Not secure	1229 (39.0)
Meal plan	
Yes	1186 (35.7)
No	2135 (64.3)
First Generation	
No	4783 (73.2)
Yes	1755 (26.8)
Pell eligible	
No	4995 (76.4)
Yes	1543 (23.6)
Number of dependents	
0	2988 (91.8)
1–2	224 (6.9)
3 or more	43 (1.3)
Annual income	
0–5000	3212 (49.4)
5000–10,000	857 (13.2)
10,000–15,000	447 (6.9)
15,000–20,000	265 (4.1)
Above 20,000	967 (14.9)

**Table 3:** Food and nutrition insecurity by student demographics.

Food Insecurity				Nutrition Insecurity			
Predictors	Mean	SD	*p* Value	Predictors	Mean	SD	*p* Value
Marital Status			0.005 *	Marital Status			<0.001 *
Single/not married (*n* = 5883)	2.42	3.06		Single/not married (*n* = 2871)	2.95	0.99	
Married or Domestic Partnership (*n* = 547)	2.03	2.84		Married or Domestic Partnership (*n* = 306)	3.15	0.96	
Annual Income			<0.001 *	Annual income			<0.001 *
0–5000 (*n* = 3212)	2.32	3.07		0–5000 (*n* = 1502)	2.99	0.98	
5000–10,000 (*n* = 857)	2.76	3.13		5000–10,000 (*n* = 446)	2.84	1.04	
10,000–15,000 (*n* = 447)	3.2	3.22		10,000–15,000 (*n* = 232)	2.77	1.1	
15,000–20,000 (*n* = 265)	3.49	3.17		15,000–20,000 (*n* = 159)	2.83	1.03	
>20,000 (*n* = 967)	2.06	2.81		>20,000 (*n* = 598)	3.13	0.89	
Post hoc: 5000–10,000 vs. >20,000			<0.001 *	Post Hoc: 10,000–15,000 vs. >20,000			<0.001 *
10,000–15,000 vs. >20,000			<0.001 *	15,000–20,000 vs. >20,000			0.009 *
15,000–20,000 vs. >20,000			<0.001 *				
No of dependents			<0.001 *	No of dependents			0.011 *
0 (*n* = 2988)	2.41	3.07		0 (*n* = 2883)	2.99	0.98	
1–2 (*n* = 224)	3.07	3.39		1–2 (*n* = 220)	2.88	1.05	
3 or more (*n* = 43)	3.65	3.72		3 or more (*n* = 42)	2.58	1.23	
Post hoc: 0 vs. 1–2			0.006 *	Post Hoc: 0 vs. 3 or more			0.021 *
0 vs. 3 or more			0.025 *				
Meal plan			<0.001 *	Meal plan			0.857
No (*n* = 2135)	2.85	3.24		No (*n* = 2063)	2.97	1	
Yes (*n* = 1186)	1.79	1.79		Yes (*n* = 1142)	2.97	0.98	
First-generation student			<0.001 *	First-generation student			<0.001 *
No (*n* = 4783)	2.01	2.83		No (*n* = 2411)	3.09	0.92	
Yes (*n* = 1755)	3.39	3.35		Yes (*n* = 800)	2.61	1.12	
Employment			<0.001 *	Employment			0.002 *
No (*n* = 2867)	2.02	2.89		No (*n* = 1268)	3.04	0.96	
Yes (*n* = 3478)	2.68	3.12		Yes (*n* = 1878)	2.93	1.01	
Pell eligible			<0.001 *	Pell eligible			<0.0018 *
No (*n* = 4995)	1.98	2.82		No (*n* = 2521)	3.08	0.94	
Yes (*n* = 1543)	3.66	3.37		Yes (*n* = 690)	2.59	1.09	
Gender			<0.001 *	Gender			0.099
Female (*n* = 4135)	2.47	3.05		Female (*n* = 2196)	2.95	0.98	
Male (*n* = 2403)	2.22	3.03		Male (*n* = 1015)	3.01	1.02	
Housing Security			<0.001 *	Housing Security			<0.001 *
Secure (*n* = 1924)	1.39	2.4		Secure (*n* = 1908)	3.26	0.82	
Not secure (*n* = 1229)	4.13	3.31		Not secure (*n* = 1212)	2.51	1.06	
Race			<0.001 *	Race			0.061
NRA (*n* = 743)	2.47	2.91		NRA (*n* = 377)	2.79	1.01	
Latin (*n* = 1050)	3.36	3.34		Latino (*n* = 487)	2.66	1.08	
Native American (*n* = 4)	4.25	4.42		Native American (*n* = 1)	3.5	-	
Asian American (*n* = 852)	2.35	3.06		Asian American (*n* = 369)	2.91	1.04	
Black (*n* = 464)	3.83	3.47		Black (*n* = 185)	2.53	1.05	
White (*n* = 3089)	1.79	2.7		White (*n* = 1629)	3.17	0.88	
Multi (*n* = 262)	2.61	3.18		Multi (*n* = 132)	2.92	1.12	
Post hoc: Native American vs. White			<0.001 *				

Note: * *p* < 0.05.

**Table 4:** Regression estimates for food and nutrition insecurity.

	Food Insecurity				Nutrition Insecurity			
Demographics	Exp (B)	Sig.	95% CI		Exp (B)	Sig.	95% CI	
			Lower	Upper			Lower	Upper
No of dependents (ref 0 dependents)								
1–2	1.61	0.017 *	1.09	2.38	1.81	0.007	1.17	2.78
3 or more	1.69	0.206	0.75	3.78	2.91	0.017	1.21	6.97
Housing security (ref Secure)								
Not secure	6.01	<0.001 *	5.02	7.2	4.04	<0.001 *	3.29	4.97
Employment (ref No)								
Yes	1.12	0.084	0.98	1.47	1.08	0.524	0.85	1.36
Gender (ref Male)								
Female	1.02	0.839	0.85	1.23	1.05	0.64	0.85	1.31
Race (ref NRA)								
Latino	1.24	<0.001 *	0.87	1.75	0.88	0.534	0.6	1.31
Native American		0.185			0	1	0	
Asian American	0.65	0.22	0.45	0.95	1.14	0.524	0.76	1.72
Black	1.56	<0.001 *	1	2.44	1.25	0.359	0.78	2.02
White	0.56	<0.001 *	0.43	0.77	0.6	0.003 *	0.43	0.84
Multi	0.87	0.786	0.53	1.42	1.04	0.879	0.6	1.81
First-generation student (ref No)								
Yes	1.61	<0.001 *	1.29	2.01	1.91	<0.001 *	1.51	2.43
Pell eligible (ref No)								
Yes	2.06	<0.001 *	1.63	2.61	1.19	0.179	0.92	1.55
Annual income (ref > 20 k)								
0–5 k	1.52	0.013 *	1.14	2.04	1.75	0.002 *	1.23	2.49
5–10 k	1.74	0.014 *	1.26	2.41	1.87	0.001 *	1.27	2.76
10–15 k	1.97	<0.001 *	1.34	2.88	1.99	0.002 *	1.29	3.08
15–20 k	1.71	0.008 *	1.12	2.59	1.93	0.008 *	1.19	3.13
Age (ref 18–30 years)								
31–40	0.88	0.533	0.58	1.33	0.65	0.115	0.38	1.11
41 and above	0.85	0.583	0.47	1.53	0.25	0.003 *	0.1	0.64
Marital status (ref Single/not married)								
Married or domestic partnership	0.71	0.063	0.49	1.02	0.68	0.08	0.44	1.05

Note: * *p* < 0.05.

## Data Availability

The original contributions presented in this study are included in the article. Further inquiries can be directed to the corresponding author.
